# Femoral Stem Subsidence and its Associated Factors after Cementless Bipolar Hemiarthroplasty in Geriatric Patients

**DOI:** 10.5704/MOJ.2103.010

**Published:** 2021-03

**Authors:** A Gema, KA Irianto, R Setiawati

**Affiliations:** 1 Department of Orthopaedic and Traumatology, Universitas Airlangga, Surabaya, Indonesia; 2 Department of Radiology, Universitas Airlangga, Surabaya, Indonesia

**Keywords:** harris hip score, hemiarthroplasty, post-operative pain, stem subsidence

## Abstract

**Introduction:**

Early femoral stem subsidence has been a concern as a predictor of the beginning of implant loosening, especially on cementless hip arthroplasty implants. This study aimed to determine the factors that affect femoral stem subsidence and outcome following hemiarthroplasty in the geriatric population.

**Materials and Methods:**

This is a retrospective study of 179 patients who underwent cementless bipolar hemiarthroplasty during the 2011-2019 period at an orthopaedic and traumatology hospital. Data on the patient's demography, pre-operative American Society Anaesthesiologist (ASA) score, body mass index (BMI), canal flare index (CFI), Dorr classification, and stem alignment were obtained. The primary outcomes were post-operative femoral stem subsidence, post-operative pain, and functional outcome using Harris Hip Score (HHS). Statistical analysis was conducted to identify risk factors associated with the primary outcome.

**Results:**

The mean femoral stem subsidence was 2.16 ±3.4 mm. The mean post-operative Visual Analog Score (VAS) on follow-up was 1.38 ± 1. Mean HHS on follow-up was 85.28±10.3. American Society Anaesthesiologist score 3 (p = 0.011, OR = 2.77) and varus alignment (p=0.039, OR = 6.963) were related to worse stem subsidence. Otherwise, neutral alignment (p = 0.045 and OR = 0.405) gave protection against femoral stem subsidence. The female gender (p = 0.014, OR 2.53) was associated with postoperative pain onset. Neutral alignment had significant relationship with functional outcomes (p = 0.01; OR 0.33).

**Conclusion:**

A higher ASA score and varus stem alignment were related to a higher risk of femoral stem subsidence. Meanwhile, neutral stem alignment had a protective effect on the femoral stem subsidence and outcome.

## Introduction

Low bone density in geriatric patients with osteoporosis can cause fractures, even with minimal energy^1^. The prevalence of femoral neck fractures had increased, owing to the rapidly developing geriatric population and longer life expectancy. It was estimated that by 2030 there would be 300,000 hip fractures cases annually in the USA^2^. A study conducted by Irianto *et al*^3^ showed that femoral neck fractures were the second most common geriatric fracture, which occurs approximately 24%, followed by fractures in the vertebra (25%). The mortality rate in patients with femoral neck fractures per year is 25-30%^4^.

The femoral neck fracture healing process in the geriatric population is slower^3,4^. The anatomy of the femoral neck and intracapsular fracture make it difficult for proper bone healing. Hip hemiarthroplasty is one of the most common surgery performed for femoral neck fracture other than total hip arthroplasty. Compared to total hip arthroplasty, the procedure is relatively faster, with less bleeding and earlier weight-bearing mobilisation^5^. There are no significant differences between the advanteges of cementless and cemented stem, however a comparison study reported that there are more respiratory or cardiovascular complications and longer duration of surgery in cemented stem^6^.

A study by Iamthanaporn *et al*^7^ found the most common etiology for hemiarthroplasty revision were aseptic loosening (49,6%) followed by infection (22,6%) and acetabular erosion (15%). Early stem migration is a predictive factor for aseptic loosening in the first and second years after hip arthroplasty surgery. Femoral stem subsidence is the distalisation of the stem relative to the major trochanter.

Two previous studies about cementless hemiarthroplasty in femoral neck patients found different results. Kabelitz *et al*^8^ found 12% intra-operative fractures and a low subsidence rate (5%). Furthermore, a study by Choi *et al* found no subsidence^6^. These conflicting results also support the notion of how studies of femoral stem subsidence in hemiarthroplasty are still limited, especially in developing countries^9^. This study aimed to determine clinical and radiographic risk factors affecting stem subsidence manifestation, post-operative pain, and functional outcome in geriatric patients with femoral neck fractures treated with cementless bipolar hemiarthroplasty.

## Material and Method

**Fig. 1:**
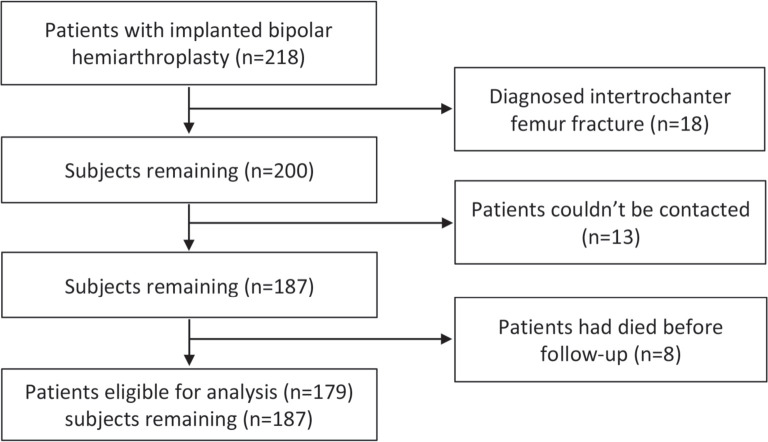
Flowchart of the patient selection process.

This is a retrospective study of femoral neck fracture in geriatric patients undergoing cementless bipolar hemiarthroplasty at an orthopaedic and traumatology hospital between 2011-2019. This study had been reviewed and received ethical clearance from Internal Institutional Review Board (No.292/EC/KEPK/FKUA/2019). All surgeries were performed by the senior author using the posterior approach. The implant used was standardised titanium-niobium Quadra-H stem [Medacta International, Castel San Pietro, Switzerland] with hydroxyapatite coating. The samples were collected from medical records of geriatric patients with femoral neck fractures who underwent cementless bipolar hemiarthroplasty and met the inclusion and exclusion criteria. The inclusion criteria were (1) patients with displaced femoral neck fractures with age ≥ 60 years, (2) pre-operative active condition and independently mobile, and (3) treated with cementless bipolar hemiarthroplasty surgery. Meanwhile, exclusion criteria were: (1) incomplete medical record, (2) pre-operative history of intertrochanter fractures, (3) and the patient had died during the evaluation. Demographic data including age, race, sex, body mass index (BMI), radiological findings, post-operative pain, and functional outcomes were recorded (Fig. 1). In terms of Body Mass Index (based on Asia-Pacific Population) classification, patients were classified as underweight (<18.5), normal (18.5-22.9), overweight (2327.49), and obesity (≥27.5).

**Fig. 2:**
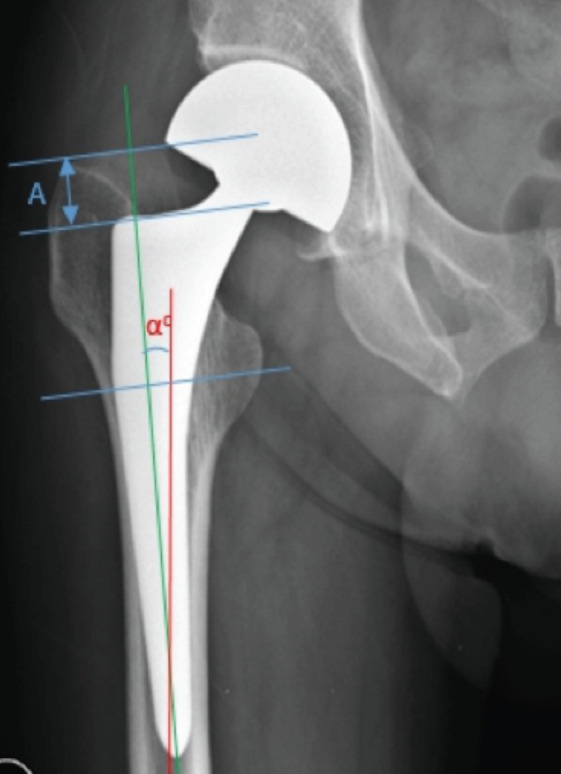
Anteroposterior post-operative radiograph of a right hip showing determination of femoral stem subsidence and alignment of the femoral stem.

Stem subsidence was evaluated from anteroposterior hip or pelvis plain radiograph (line A in Fig. 2) by measuring the difference in distance from the trochanter major's peak to the stem shoulder perpendicular to the axis of the femur stem at a certain period^10^. The anteroposterior pelvic or hip plain radiograph was taken in supine position, centering on the symphysis and 120cm distance of the film focus. The femoral stem subsidence was divided into two groups following the Al-Najjim study: below and ≥ 3mm^11^. Stem alignment was the stem's position against the longitudinal axis of the femur (Fig. 2). Alignment degree was carried out by measuring the angle from the axis of the femur stem to the longitudinal axis of the post-operative femur^12,13^. The alignment was categorised into three groups: neutral, varus, and valgus.

**Fig. 3:**
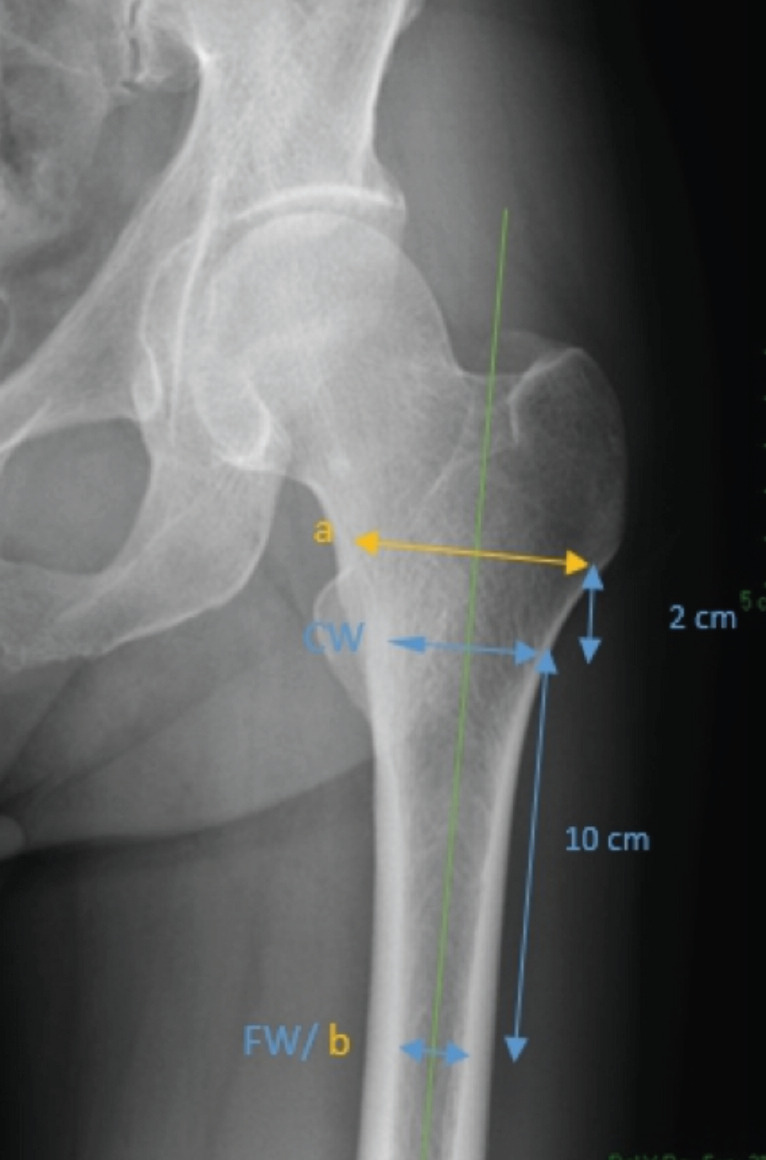
Anteroposterior pre-operative radiograph of a right hip showing determination CFI and Dorr score.

**Fig. 4:**
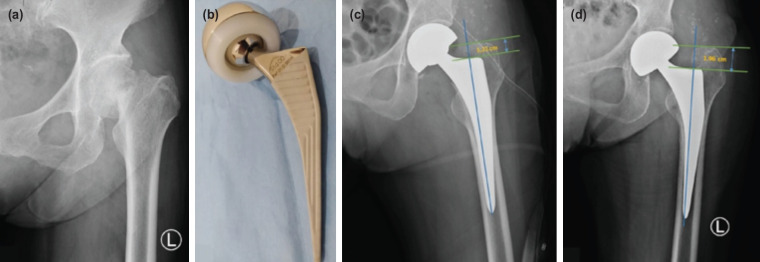
(a) Example of measured femoral stem subsidence in a female, 79 years old. Pre-operative radiograph. (b) Implants that was used in this study. (c) Post-operative radiograph. (d) Radiograph taken during follow-up (three months).

The morphology of the proximal femur was described as the structural quality of the bones divided by three categories based on the Dorr classification: A (<0.5), B (0.5-0.75), and C (0.75). The Dorr score was obtained by calculating the ratio between the medulla canal diameter at a distance of 10cm below the trochanter minor and the medulla canal as high as the trochanter minor (Dorr = FW / CW) (Fig. 3). Anatomy of the proximal femur was categorised into three groups by calculating the Canal Flare Index (CFI). Based on Nguyen *et al* 's study^14^, CFI was measured by dividing the femur canal diameter at 2cm above the midpoint of the minor trochanter with the femur canal diameter at 10cm below the trochanter minor (CFI = a / b). This measurement is used to classify the medullary canal for femoral component selection in total hip arthroplasty, is also reported to be associated with bone quality and osteoporosis. CFI was classified as stovepipe (< 3), normal (3 - 4.7), and champagne-fluted (> 4.7)^14^. A femoral stem subsidence evaluation was done shortly after surgery and at the latest follow-up (at least six weeks after surgery) (Fig. 4)^13^. CFI and Dorr were measured pre-operatively. The alignment was measured from the first post-operative plain radiograph (within one week of surgery). Radiological measurements on each photo were calibrated to correct the scale. Radiological measurement was done blindly by two musculoskeletal consultant radiologists. Inter-observer reliability based on Cohen’s kappa value was 0.82 (95% CI= 0.74-0.92; p<0.05).

The American Society of Anaesthesiologists (ASA) score indicates the patient's pre-operative comorbidity conditions that are divided into five classes: ASA scores 1 (healthy patient), 2 (patient with the mild systemic disease), 3 (patient with the severe systemic disease not incapacitating), 4 (patient with incapacitating systemic disease), and 5 (a moribund patient)^15^. Post-operative pain was evaluated using the Visual Analog Score (VAS), which was divided into two groups, no pain (VAS 0) and pain (VAS 1-5), due to the absence of patients who complained of severe pain. In this study, functional outcome measurement used the Harris Hip Score (HHS) was used to measure functional outcome. which was evaluated in patients using four domains: pain, function, range of motion (ROM), and presence or absence of deformity after bipolar hemiarthroplasty procedures. HHS in this study was divided into two groups, namely poor (HHS <80) and good (HHS 80-100). Post-operative pain and HHS were measured at the last follow-up.

Statistical analysis such as Chi square test or Kruskal Wallis test were used as appropriate based on each variables. The variables used in the multivariate logistic analysis were selected from the univariate analysis with p value < 0.25. Multivariate analysis with logistic regression was performed to determine the risk factors influencing femoral stem subsidence the most, post-operative pain, and HHS. A value of p <0.05 was significant. SPSS 25.0 program was used for all statistical analysis [SPSS Inc., Chicago, USA]

## Results

There were 218 patients who underwent bipolar hemiarthroplasty surgery, and 179 patients met the inclusion criteria. The mean follow-up time was 42 months, and the average age was approximately 74.5 ±8.1 years. One hundred and forty-one (78.8%) patients came on the last follow-up with post-operative pain, and 38 (21.2%) patients came with no post-operative pain. The mean of VAS on follow-up was 1.38 ± 1. In terms of HHS classification, 138 (77.1%) patients were in good functional outcome, and 41 (22.9%) patients were in poor functional outcome. The mean of HHS on follow-up was 85.28±10.3. The mean of femoral stem subsidence was 2.16 ±3.4mm. There was no revision surgery nor perioperative mortality in this study.

There were significant correlation between patient’s age, ASA score, stem alignment and HHS with the subsidence in stem (p = 0.035, p = 0.036, p = 0.001 and p = 0.001) Table I. Besides, there was no significant relationship between sex, BMI, CFI, Dorr Score and post-operative pain with stem subsidence (p = 0.700, p = 0.845, p = 0.142, p = 0.879 and p= 0.313, respectively). This study showed that female patients associated with more post-operative pain than male patients (p = 0.012) Table II. There is also significant relationship between HHS and post-operative pain (p = 0.041). There were no significant differences between postoperative pain with age, ASA score, BMI, Dorr, CFI, and alignment (p > 0.05). There were significant relationships between alignment and femoral stem subsidence with postoperative functional outcome (p = 0.001 and <0.001, respectively) Table II.

**Table I:** Demographics of patients

	Frequency (n)	Percentage (%)
Age		
60-74	90	50.27
75-90	87	48.61
>90	2	1.12
Sex		
Male	55	30.7
Female	124	69.3
Race		
Malay	28	15.6
Chinese	151	84.4
ASA Score		
ASA score 1	8	4.46
ASA score 2	103	57.54
ASA score 3	68	37.98
BMI		
<18.5 (Underweight)	32	17.9
18.5 - 22.9 (Normal)	77	43
23 - 27.49 (Overweight)	35	19.6
≥27.5 (Obesity)	35	19.6
Alignment		
Valgus	33	18.4
Normal	139	77.7
Varus	7	3.9
CFI		
Stovepipe	27	15.1
Normal	125	69.8
Champagne fluted	27	15.1
DORR		
A	149	83.24
B	30	16.75
C	0	0

Abbreviations: BMI = Body Mass Index, CFI = Canal Flare Index, ASA = American Society of Anaesthesiologists

**Table II:** Characteristics of patients and its relationship with stem subsidence, post-operative pain and HHS

Characteristic	<3mm	Subsidence ≥ 3mm	P Value	no pain	Post-operative pain pain	P value	Low	HHS Good	P Value
Age			0.035+			0.471+			0.465+
60-74	77 (43%)	13 (7.3%)		22 (12.3%)	68 (38%)		18 (10%)	72 (40.2%)	
75-90	61 (34.1%)	26 (14.5%)		16 (8.9%)	71 (39.7%)		22 (12.3%)	65 (36.3%)	
>90	(1.1%)	0(0%)		0(0%)	(1.1%)		(0.6%)	(0.6%)	
Sex		0.700*			0.012*			0.818*	
Male	44 (24.6%)	11 (6.2%)		18 (10.1%)	37 (20.6%)		12 (6.7%)	43 (24%)	
Female	96 (53.6%)	28 (15.6%)		20 (11.2%)	104 (58.1%)		29 (16.2%)	95 (53.1%)	
Race			0.122*			0.978*			0.774*
Malay	25 (14%)	(1.7%)		(3.3%)	22 (12.3%)		(3.9%)	21 (11.7%)	
Chinese	115 (64.2%)	36 (20.1%)		32 (17.9%)	119 (66.5%)		34 (19%)	117 (65.4%)	
BMI			0.845*			0.790*			0.837*
<18.5	24 (13.4%)	(4.5%)		(3.3%)	26 (14.6%)		(3.9%)	25 (14%)	
18.5 -22.9	62 (34.6%)	15 (8.4%)		19 (10.6%)	58 (32.4%)		16 (8.9%)	61 (34%)	
23 -27.49	28 (15.7%)	(3.9%)		(3.3%)	29 (16.2%)		(4.5%)	27 (15.1%)	
>27.5	26 (14.5%)	(5%)		(3.9%)	28 (15.7%)		10 (5.6%)	25 (14%)	
ASA score			0.036*			0.051*			0.712*
1	(4.5%)	0(0%)		(2.2%)	(2.2%)		(0.6%)	(3.9%)	
2	85 (47.5%)	18 (10%)		24 (13.4%)	79 (44%)		23 (12.8%)	80 (44.7%)	
3	47 (26.3%)	21 (11.7%)		10 (5.6%)	58 (32.4%)		17 (9.5%)	51 (28.5%)	
CFI		0.142*			0.185*			0.237*	
Stovepipe	20 (11.2%)	(3.9%)		(1.7%)	24 (13.4%)		(4.5%)	19 (10.6%)	
Normal	95 (53.1%)	30 (16.7%)		28 (15.6%)	97 (54.2%)		30 (16.8%)	95 (53.1%)	
Champagne fluted	25 (14%)	(1.1%)		(3.9%)	20 (11.2%)		(1.7%)	24 (13.4%)	
Dorr			0.879*			0.757*			0.678*
A	116 (64.8%)	33 (18.4%)		31 (17.3%)	118 (65.9%)		35 (19.5%)	114 (63.7%)	
B	24 (13.4%)	(3.4%)		(3.9%)	23 (12.9%)		(3.4%)	24 (13.4%)	
C	0(0%)	0(0%)		0(0%)	0(0%)		0(0%)	0(0%)	
Alignment			0.001*			0.778*			0.001*
Valgus	22 (12.3%)	11 (6.2%)		(4.5%)	25 (14%)		12 (6.7%)	21 (11.7%)	
Normal	116 (64.8%)	23 (12.8%)		28 (15.6%)	111 (62%)		22 (12.3%)	117 (65.4%)	
Varus	(1.1%)	(2.8%)		(1.1%)	(2.8%)		(3.9%)	0(0%)	
HHS			0.001*			0.041*			n.a
Good	120 (67%)	18 (10.1%)		34 (19%)	104 (58.1%)		n.a	n.a	
Low	20 (11.2%)	21 (11.7%)		(2.2%)	37 (20.7%)				
Subsidence			n.a			0.313*			0.001*
<3mm	n.a	n.a		32 (17.9%)	108 (60.3%)		20 (11.2%)	120 (67%)	
>3mm				(3.4%)	33 (18.4%)		21 (11.7%)	18 (10.1%)	

Abbreviations: n.a = not available; HHS = Harris Hip Score; BMI = Body Mass Index, CFI = Canal Flare Index, ASA = American Society of Anaesthesiologists

* Chi square test

+ Kruskal-Wallis Test

The results of multivariate analysis are listed in Table III. Alignment and ASA score remained significant risk factors for the subsidence. Neutral alignment gave protective effects from the femoral stem subsidence with p = 0.045 and OR 0.405 (CI = 0.16-0.98). Meanwhile, varus alignment associated with higher femoral stem subsidence with p = 0.039 and OR = 6.96 (CI = 1.09-44.12). ASA score 3 was associated with higher femoral stem subsidence with p = 0.011 and OR = 2.771 (CI = 1.25-6.10). The female population was considered to be associated with postoperative pain with p = 0.014 and OR= 2.53 (CI = 1.215.29). Neutral alignment was correlated with better functional outcome with p = 0.01 and OR 0.329 (CI= 0.142 - 0.764).

**Table III:** Multivariate analysis results

Variables	Subsidence		Post-operative pain		HHS	
	odds (% CI)	P value	odds (% CI)	P value	odds (% CI)	P value
Sex						
Male						
Female			2.53 (1.21-5.29)	0.014*		
CFI						
Stovepipe						
Normal	1.344 (0.464-3.890)	0.586				
Champagne fluted	0.284 (0.045-1.808)	0.183				
Alignment						
Valgus	0.405 (0.167-0.980)	0.045*			0.329 (0.142-0.764)	0.01*
Normal	6.963 (1.099-44.122)	0.039*			4.375(0.733-26.116)	0.105
Varus						
ASA score						
ASA 1 and 2						
ASA 3	2.771 (1.258-6.104)	0.011*				

Abbreviations: CFI = Canal Flare Index; ASA = American Society of Anesthesiologists

* statistically significant

## Discussion

In this study, we found that among 179 patients, more than 60% of them are female patients. This is in line with the study by Iglesias *et al*^4^, showing that women suffer more fractures of the femoral neck. This could be related to menopause causing decline in bone density. Decreasing bone density could influence neck-shaft angle and hip axis length resulting in a higher risk of a femoral neck fracture^16^. A study conducted by Novicoff and Saleh showed that women also had worse hip degeneration and cartilage loss than men^17^.

Moreover, a recent study showed that female patients were significantly associated with post-operative pain. This is in line with the Mannion *et al*^18^ research that women had a higher Western Ontario and McMaster Universities Osteoarthritis Index (WOMAC) post-operative pain than men, although it was not significant. Women also had lower pain thresholds, making them more sensitive to pain compared to men. Thus, there could be excess pain and poorer functional outcome in women after surgery^17^.

Most of the patients in this study experienced <3mm subsidence in stem (78.2%). The mean of subsidence was 2.16mm (SD 3.4), which was lower than what was found in the previous study of 2.9mm (SD 2.7)^11^ and 3.9mm (SD 2)^8^. This result could be affected by the greater cortical thickness and higher volumetric bone mineral density (BMD) in Asia patients than white patients^13^. This study showed association between patient age and stem subsidence. Furthermore, the number of geriatric patients in the age 75-90 years with femoral stem subsidence ≥3mm (14.5%) was greater than patients with the group of age 60-74 years (7.3%). A study by Song *et al*^19^ showed similar results where age showed a significant effect on stem subsidence. Large stem subsidence (7.15-19.38mm) was found at 62-89 years. Bone stock could influence femoral stem subsidence. Nazari-Farsani *et al*^20^ found that older women with low bone mineral density were at risk of developing femoral stem subsidence (OR=6.7).

Anatomy and the proximal femur morphology may affect femoral stem subsidence in patients who have undergone arthroplasty surgery. A suitable match of a femoral stem with bone will optimise initial torque stability, thereby improve bone growth and reduce fibrous growth^13^. Dorr type C was associated with a lower T-score than type A, implying that bone density in Dorr C was lower than type A^21^. Other studies said that the Dorr type C was correlated with lower cortical indices, femoral stem subsidence, and higher risk intra-operative fractures^14,19^. However, this study showed no significant relationship between Dorr type and CFI with femoral stem subsidence in patients.

The comorbidity assessment using ASA scores showed a correlation to various factors, including the length of stay, the severity of complications, and the treatment's total cost. A higher ASA score was associated with a higher risk of post-operative complications and mortality rates^15,22^. This study showed a significant relationship between ASA score 3 with the stem subsidence (p = 0.011, OR = 2.77). Kinjo *et al*^23^ found a similar result in their cohort study that pre-operative pain, pre-operative opioids, higher ASA scores, and female patients were associated with higher post-operative pain. The patient's pre-operative comorbidity could be cardiac disease, respiratory disease, obesity, liver disease, or other systemic diseases. A recent study found that among 68 patients with ASA score 3, 60.3% had comorbid hypertension, 20.5% had diabetes mellitus, 14.7% had obesity, and 14.7% had chronic renal disease. Bone condition changes associated with hyperglycemia or chronic renal disease could affect bone quality, delaying bone-ingrowth fixation at the bone-implant interface, the bone healing process, vascularisation, and subsequent mechanical stability^24^. In their study, Chen *et al*^22^ found that a higher ASA score was associated with older patients that had low bone density (79.39 ±7.85 years old). Those results might explain our finding that the ASA score affected femoral stem subsidence.

Multivariate analysis results showed that the neutral alignment had a protective effect on femoral stem subsidence and functional outcome. Meanwhile, the varus alignment group showed a significant effect on stem subsidence. This result is in line with the study conducted by de Beer and Zang, where the majority of patients had neutral stem alignment^25^. A study by Ries *et al*^13^ showed no significant relationship between stem alignment and stem subsidence. However, Kutzner *et al*^26^ mentioned that stem valgus alignment increased the number of stem subsidence but did not affect clinical outcomes. The alignment that was too varus increase the offset and increase the lever arm^26^. This could escalate strain and stress on the medial side of the proximal femur and the area at the distal end of the stem, so that it initiated periprosthetic fracture both during and post-operatively^25^. Therefore, we proposed that femoral alignment should be placed properly intra-operative.

The functional outcomes of most patients in this study after cementless bipolar hemiarthroplasty showed a good result (HHS 80-100) at 77,1%. Moreover, there was a significant relationship between stem subsidence and HHS. Femoral stem subsidence could lead to a decline in functional outcomes like dislocation, leg length discrepancy, and pain. Early stem subsidence was associated with a higher risk of inadequate secondary integration resulting in micromotion and fibrous fixation^13^. Most osteointegration is achieved in early 4-12 weeks, and cementless implants are highly dependent on bone osteointegration with this implant's surface. During walking exercise, the load was transferred from the implant to the bone, pushing the bone further and cause subsidence in the stem through mechanical stability^11^. According to Al-Najjim *et al*^11^ and Song *et al*^19^ stem subsidence of more than 3mm was associated with poor outcomes and complications such as loosening and fracture of the stem. The radiological signs of loosening or failure in the implant include stem subsidence, osteolysis, bone resorption in femoral calcar, osteopenia, and radiolucent lines around the implant^27^.

De Nies and Malcolm's research supports this study result. They found a significant correlation between HHS and pain levels as measured by VAS28. This can be explained that patients with minimum or zero pain can carry out their daily activities to gain better quality of life. Among the 141 patients who complained of post-operative pain, 33 (23.4%) experienced subsidence of more than 3mm. Of these 33 patients, 5 (15.1%) had stovepipe morphology, 2 (6.06%) had champagne-fluted morphology, 12 (36.3%) with a BMI of >23, and 15 (45.4%) patients had ASA score 3. Early weight-bearing in patients with poor proximal femur morphology, bone density, pre-operative conditions, overweight, and intra-operative factors could trigger early migration. In addition, the cause of post-operative pain with implant loosening was considered the presence of micromotion on the bone and implant interfaces, stress transfer imbalance, and irritation in endosteum periosteum^27^.

A previous study by Nishi *et al*^9^, reported a lower rate of revision surgery after cementless hemiarthroplasty. Another study by Kabelitz *et al*^8^ supported this suggestion with the low rate of early subsidence. Despite that, some articles also reported about higher volume of blood loss after surgery^6^. Another concern about cementless hemiarthroplasty is a higher rate of complications, namely intra-operative fractures in osteoporotic bone8 which didn’t occur in this study.

This study has limitations. The design of this study is retrospective, which subjects to biases and confounding which can influence the result. The other limitations are relatively small number of patients, and the duration of the patient's observation was not long enough. Further studies are needed to assess the evidence of aseptic loosening; more comprehensive study follow-up and multiple examination to see early and late-onset subsidence and its outcome. Nevertheless, this study highlights the importance of preventing femoral stem subsidence by achieving good stem fixation in the femoral diaphysis during the intra-operative period. Post-operative functional outcomes are closely related to intra-operative management, post-operative rehabilitation, and pre-operative comorbid factors.

## Conclusion

Higher ASA scores and varus alignment of the stem were associated with a higher risk of femoral stem. Neutral stem alignment of the femoral stem gave the protective effect of stem subsidence and better functional outcome. The use of cementless femoral stem is not recommended in elderly patients in treating the femur neck fracture.

## References

[1] Sandhu HS, Dhillon MS, Jain AK (2008). Femoral neck fractures.. Indian J Orthop..

[2] Brox WT, Roberts KC, Taksali S, Wright DG, Wixted JJ, Tubb CC (2015). The American Academy of Orthopaedic Surgeons Evidence-Based Guideline on Management of Hip Fractures in the Elderly.. J Bone Joint Surg Am..

[3] Irianto KA, Rianto D, Sukmajaya WP, Alina O (2019). Geriatric fractures in single Orthopedic Hospital: The role of domestic fall and comprehensive geriatric assessment.. Bali Med J..

[4] Iglesias SL, Gentile L, Vanoli F, Mangupli MM, Pioli I, Nomides REK (2017). Femoral neck fractures in the elderly: from risk factors to pronostic features for survival.. J Trauma Crit Care..

[5] Prokop A, Chmielnicki M (2016). Hemiprosthesis for femoral neck fractures in the elderly: A retrospective study of 319 patients.. Arch Trauma Res..

[6] Choi JY, Sung YB, Kim JH (2016). Comparative Study of Bipolar Hemiarthroplasty for Femur Neck Fractures Treated with Cemented versus Cementless Stem.. Hip Pelvis..

[7] Iamthanaporn K, Chareancholvanich K, Pornrattanamaneewong C (2018). Reasons for revision of failed hemiarthroplasty: Are there any differences between unipolar and bipolar?. Eur J Orthop Surg Traumatol..

[8] Kabelitz M, Fritz Y, Grueninger P, Meier C, Fries P, Dietrich M (2018). Cementless Stem for Femoral Neck Fractures in a Patient’s 10th Decade of Life: High Rate of Periprosthetic Fractures.. Geriatr Orthop Surg Rehabil..

[9] Nishi M, Okano I, Sawada T, Midorikawa N, Inagaki K (2019). Cementless Bipolar Hemiarthroplasty for Low-energy Intracapsular Proximal Femoral Fracture in Elderly East-Asian Patients: A Longitudinal 10-year Follow-up Study.. Hip Pelvis..

[10] Siepen W, Zwicky L, Stoffel KK, Ilchmann T, Clauss M (2016). Prospective two-year subsidence analysis of 100 cemented polished straight stems - A short-term clinical and radiological observation.. BMC Musculoskelet Disord..

[11] Al-Najjim M, Khattak U, Sim J, Chambers I (2016). Differences in subsidence rate between alternative designs of a commonly used uncemented femoral stem. J Orthop..

[12] Morgenstern R, Denova TA, Khan I, Carroll KM, Su EP (2019). Total hip arthroplasty utilizing an uncemented, flat, tapered stem with a reduced distal profile. Arthroplast Today..

[13] Ries C, Boese CK, Dietrich F, Miehlke W, Heisel C (2019). Femoral stem subsidence in cementless total hip arthroplasty: a retrospective single-centre study.. Int Orthop..

[14] Nguyen BN, Hoshino H, Togawa D, Matsuyama Y (2018). Cortical thickness index of the proximal femur: A radiographic parameter for preliminary assessment of bone mineral density and osteoporosis status in the age 50 years and over population.. Clin Orthop Surg..

[15] Kastanis G, Topalidou A, Alpantaki K, Rosiadis M, Balalis K (2016). Is the ASA Score in Geriatric Hip Fractures a Predictive Factor for Complications and Readmission?. Scientifica (Cairo)..

[16] Gnudi S, Sitta E, Pignotti E (2012). Prediction of incident hip fracture by femoral neck bone mineral density and neck-shaft angle: A 5-year longitudinal study in post-menopausal females.. Br J Radiol..

[17] Novicoff WM, Saleh KJ (2011). Examining sex and gender disparities in total joint arthroplasty.. Clin Orthop Relat Res..

[18] Mannion AF, Impellizzeri FM, Naal FD, Leunig M (2015). Women demonstrate more pain and worse function before THA but comparable results 12 months after surgery.. Clin Orthop Relat Res..

[19] Song JH, Jo WL, Lee KH, Cho YJ, Park J, Oh S (2019). Subsidence and perioperative periprosthetic fractures using collarless hydroxyapatite-coated stem for displaced femoral neck fractures according to Dorr type.. J Orthop Surg (Hong Kong)..

[20] Nazari-Farsani S, Vuopio ME, Aro HT. Bone Mineral Density, Cortical-Bone Thickness (2020). of the Distal Radius Predict Femoral Stem Subsidence in Postmenopausal Women.. J Arthroplasty..

[21] Glowacki J, Thornhill TS (2016). Osteoporosis and Osteopenia in Patients with Osteoarthritis.. Ortho & Rheum Open Access J..

[22] Chen LH, Liang J, Chen MC, Wu CC, Cheng HS, Wang HH (2017). The relationship between preoperative American Society of Anesthesiologists Physical Status Classification scores and functional recovery following hip-fracture surgery.. BMC Musculoskelet Disord..

[23] Kinjo S, Sands LP, Lim E, Paul S, Leung JM (2012). Prediction of postoperative pain using path analysis in older patients.. J Anesth..

[24] Tornero E, Cofan F, Reategui D, Gracia-Toledo M, Campistol JM, Riba J (2015). Outcomes of hip arthroplasty in patients with end-stage renal disease : a retrospective, controlled study.. Int J Adv Jt Reconstr..

[25] Zang J, Uchiyama K, Moriya M, Li Z, Fukushima K, Yamamoto T (2018). Long-term clinical and radiographic results of the cementless spotorno stem in japanese patients: A more than 15-year follow-up.. J Orthop Surg (Hong Kong)..

[26] Kutzner KP, Freitag T, Donner S, Kovacevic MP, Bieger R (2017). Outcome of extensive varus and valgus stem alignment in short-stem THA: clinical and radiological analysis using EBRA-FCA.. Arch Orthop Trauma Surg..

[27] Clauss M, Van Der Straeten C, Goossens M (2014). Prospective five-year subsidence analysis of a cementless fully hydroxyapatite-coated femoral hip arthroplasty component. HIP Int..

[28] de Nies F, Fidler MW (1997). Visual analog scale for the assessment of total hip arthroplasty.. J Arthroplasty..

